# The Utility of the Electromyography and Ultrasound Guidance Combination for Botulinum Neurotoxin Injection: Focus on the Added Value of Electromyography

**DOI:** 10.3390/toxins18060238

**Published:** 2026-05-22

**Authors:** Domenico Antonio Restivo, Mario Stampanoni Bassi, Rosario Marchese-Ragona, Giovanni Castelnovo, Angelo Alito, Demetrio Milardi, Stefano Masiero, Daniele Coraci

**Affiliations:** 1 Department Clinical and Experimental Medicine, University of Messina, 98122 Messina, Italy; 2 Brain Mapping Lab, Department of Biomedical, Dental Sciences and Morphological and Functional Imaging, University of Messina, 98122 Messina, Italy; alitoa@unime.it (A.A.); dmilardi@unime.it (D.M.); 3 Unit of Neurology, IRCCS Neuromed, 86077 Pozzilli, Italy; m.stampanonibassi@gmail.com; 4 Faculty of Psychology, Uninettuno Telematic International University, 00186 Rome, Italy; 5 Department of Neurosciences, University of Padova, 35122 Padova, Italy; rosario.marcheseragona@unipd.it (R.M.-R.); stef.masiero@unipd.it (S.M.); daniele.coraci@unipd.it (D.C.); 6 Neurological Unit, Chu University, 30029 Nimes, France; giovanni.castelnovo@chu-nimes.fr; 7 Department of Biomedical, Dental Sciences and Morphological and Functional Images, University of Messina, 98122 Messina, Italy

**Keywords:** botulinum neurotoxins, electromyography, EMG guidance, injection guidance, motor endplate, ultrasonography, US guidance

## Abstract

The efficacy of botulinum neurotoxin (BoNT) is strongly dependent on its accurate delivery to hyperactive muscles and, ideally, to motor endplate regions. Although guidance techniques such as electromyography (EMG) and ultrasound (US) improve injection precision, each technique provides only partial information—either functional or anatomical. Integrating these techniques could enhance targeting accuracy, optimize dose distribution, and reduce off-target effects. A structured PubMed search was performed using terms related to BoNT, spasticity/dystonia, EMG, and US. Filters included clinical trials, randomized controlled trials, meta-analyses and reviews published within the last decade. Fifty-nine studies met the inclusion criteria. The publications were predominantly in neuroscience and rehabilitation journals. Only 17 studies reported combined EMG–US guidance. These focused mainly on stroke and cervical dystonia. While EMG-US integration is a promising strategy, we emphasize the added value of EMG guidance for US approaches, which is particularly important when treating complex neurological conditions involving complex, overlapping muscle activation patterns, or when targeting structures that are inaccessible to conventional imaging techniques. The EMG-US integrated approach is a promising strategy for optimizing BoNT therapy by combining structural visualization with real-time functional assessment. Despite its promising advantages in terms of accuracy and dose optimization, its clinical adoption is limited by a lack of high-quality evidence.

## 1. Introduction

Botulinum neurotoxin (BoNT) treatment has become a key therapeutic intervention for various clinical conditions involving muscle hyperactivity, such as spasticity and dystonia [1,2,3]. The precision with which BoNT injections are targeted is directly related to the efficacy of the treatment [4,5,6]. For this reason, selecting an appropriate guidance methodology is a critical decision that can affect procedure accuracy, efficiency and patient comfort, and consequently therapeutic success. Several techniques have emerged in this field, each with its own advantages, limitations, and optimal applications. The most widely used methodological approaches for targeting BoNT injections are anatomical landmarks, electromyography (EMG) guidance and ultrasound (US) guidance [7].

The anatomical landmark technique uses surface landmarks, muscle/bone palpation and passive or active range of motion to identify target muscles. This method is the most accessible, but potentially the least precise, as it cannot visualize individual muscles or account for anatomical variations. Moreover, this technique has very limited utility for localizing deep or small muscles [7]. In a systematic review comparing injection accuracy in spasticity management, Picelli et al. demonstrated that anatomical landmark-based injections achieved target muscle accuracy rates ranging from 42% to 95%, depending on muscle size and depth. Superficial, larger muscles, including the gastrocnemius muscle, showed higher accuracy with palpation alone, while deeper muscles, such as the flexor digitorum profundus, showed substantially reduced precision. This variability underscores the limitations of surface anatomy in guiding injections to certain anatomical targets [7].

EMG guidance uses electromyographic recordings to confirm the position of the needle within electrically active muscles, providing functional confirmation of the location of the target. The rationale for EMG guidance goes beyond simple anatomical localization, providing functional confirmation of needle placement. This technique enables real-time assessment of muscle activation patterns, allowing the activity of single muscles in close anatomical proximity to be isolated. It also permits dynamic differentiation between primary pathological muscle hyperactivity and secondary compensatory responses, which can be highly relevant in the treatment of dystonia [7,8]. Furthermore, EMG allows precise needle positioning within the motor endplate region, where acetylcholine receptors are concentrated, thus maximizing the therapeutic efficacy of BoNT [9,10]. Indeed, it has been demonstrated that, since BoNT exerts its effect at the level of the presynaptic cholinergic nerve terminal, delivering the toxin directly to areas with a high concentration of motor endplates may enhance its therapeutic efficacy while potentially reducing the total dose required, minimizing spread to adjacent muscles and consequently immunogenic risk [9,10]. However, EMG cannot differentiate between muscles with similar innervation patterns or visualize surrounding neurovascular structures, nor can it confirm drug distribution into muscular structures. Moreover, EMG represents an invasive approach which may be potentially painful. Furthermore, EMG-guided injections into multilayered muscles may still result in inadvertent injection into adjacent unaffected muscles in 16–29% of cases [11].

The introduction of US guidance for BoNT treatment has revolutionized the injection technique by offering real-time visualization of anatomical structures and needle trajectory, as well as drug distribution. Picelli et al. demonstrated statistically significant improvement in clinical functional measures at 4 and 12 weeks after injection when comparing US-guided versus electrical stimulation-guided BoNT injections into upper limb muscles in patients with post-stroke spasticity [12]. Similarly, in patients with lower-limb spasticity, US guidance has been shown to improve the accuracy of toxin delivery to deep muscles. Moreover ultrasound-guided injections achieved greater spasticity reduction and more significant improvements in gait parameters compared with anatomical landmark-based injections [1]. In a study of a cohort of patients with post-stroke spasticity, Santamato et al. evaluated the accuracy of BoNT injections into various upper and lower limb muscles and demonstrated that US guidance improved accuracy by 35–40% for deep muscles compared to anatomical landmarks alone. The magnitude of improvement was inversely correlated with depth and size, with the greatest benefits observed for small, deep muscles in anatomically complex regions [13]. Furthermore, in patients with cervical dystonia, this treatment approach has been shown to enable more precise targeting of complex cervical muscles, reducing the total BoNT dose required while maintaining therapeutic efficacy [14].

Additionally, US guidance in BoNT administration offers advantages that extend beyond muscle localization and needle placement for injections. Real-time visualization of adjacent anatomical structures, including vessels and nerves, may reduce the risk of inadvertent injury and improve procedural safety, particularly in anatomically complex regions, in patients with altered anatomy or in pediatric populations, where the use of EMG is sometimes limited [15,16,17]. In addition, US allows dynamic assessment and can support more accurate targeting in deep or small muscles [13].

Dose optimization has important clinical implications, including reduced side effects, treatment costs and risk of antibody formation. Furthermore, in pediatric spasticity management, where weight-based guidelines necessitate strict adherence to safety limits, US-guided, volume-adjusted dosing has demonstrated usefulness [18,19]. Kwon et al. reported that US-guided muscle volume assessment in children with cerebral palsy facilitated more precise dose individualization, optimizing the therapeutic index. Visualizing the drug distribution in real time further enhances dose efficiency by confirming adequate muscle penetration and distribution [19]. However, this injection procedure cannot evaluate muscular hyperactivity, which is a major component of several neurological conditions including spasticity and dystonia, nor can it distinguish between primary and secondary compensatory muscular activity in dystonia [7,8]. Furthermore, due to attenuation of sound waves by overlying structures, complex three-dimensional anatomy and limited acoustic windows imposed by bony structures and air-filled viscera, US presents significant limitations when accessing deep cervical muscles or targeting laryngeal and/or pharyngeal muscles [20].

Thus, although EMG and US each have specific limitations when used individually, their combined use may be especially beneficial when treating conditions characterized by overlapping muscle activation patterns, or when targeting structures that are inaccessible via surface anatomical landmarks and conventional imaging techniques. Combining US with EMG guidance may offer synergistic advantages in specific clinical scenarios.

This hybrid approach leverages the complementary strengths of anatomical and physiological localization methods. Integrating EMG with US guidance can be useful when treating complex movement disorders where different muscle groups may be abnormally overactivated, contributing to abnormal posture. Combining these two injection procedures enables both precise anatomical targeting and confirmation of muscle activity patterns.

This review aims to examine the evolving role of integrating US and electromyographic guidance for BoNT treatment. Combining these two techniques could help to overcome the limitations of each technique by enhancing their individual strengths, thereby improving the efficacy and safety of the treatment. Although both techniques, with their specific peculiarities, significantly improve the injection accuracy on their own, their combination may further enhance the precision of targeting compared to the use of each one individually.

## 2. Results

The initial search identified 65 manuscripts [2,12,14,21,22,23,24,25,26,27,28,29,30,31,32,33,34,35,36,37,38,39,40,41,42,43,44,45,46,47,48,49,50,51,52,53,54,55,56,57,58,59,60,61,62,63,64,65,66,67,68,69,70,71,72,73,74,75,76,77,78,79,80,81,82]. Five of these were excluded due to irrelevance. Therefore, the analysis was based on 60 final studies. Regarding journal categories, the majority of these papers predominantly fell under the neuroscience category, followed immediately by the rehabilitation category [24,27,28,29,32,34,38,39,40,42,43,44,45,46,48,50,52,53,54,55,59,64,65,70,71,74,75,76,77,79,80]. The other three categories were toxinology, medicine and radiology. Regarding the year of publication, most studies were concentrated between 2015 and 2021, with a geographical distribution showing a predominance of Europe, followed by North America [23,24,27,29,30,31,32,33,35,38,39,41,42,48,49,50,51,52,53,54,56,57,58,60,61,63,64,65,66,67,69,72,74,75,76,77,79,80]. Publications from the Far East and Australia were also identified [12,14,21,22,26,28,34,40,43,44,45,46,47,70,71,73,78,81]. Single publications were also found for India, the United Arab Emirates, Chile and Brazil [36,55,61]. The most common article types were reviews (35.6%) and clinical trials (33.9%) [22,24,25,26,27,28,30,31,32,35,37,39,40,41,43,44,45,48,52,53,55,56,57,58,59,60,61,63,66,68,69,74,75,76,77,78,79,81]. Meta-analyses were present in only 8.5% of cases and originated exclusively from Asia and Europe [29,38,59,62,78,80].

**Figure 1:**
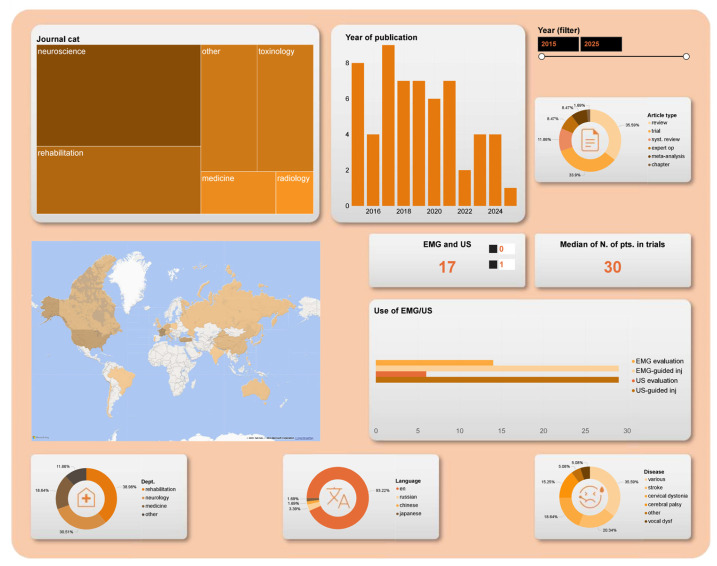
Results of the literature analysis. The following variables are shown (from left to right and from top to bottom): journal categories of the publication, year of publication, type of paper, geographical origin of the paper (based on the first author), median number of patients included in trials, number of papers where EMG or US were used for diagnosis or guiding the injection, department where the authors work, language of the paper text (en English), and diseases considered in the publications.

**Figure 2:**
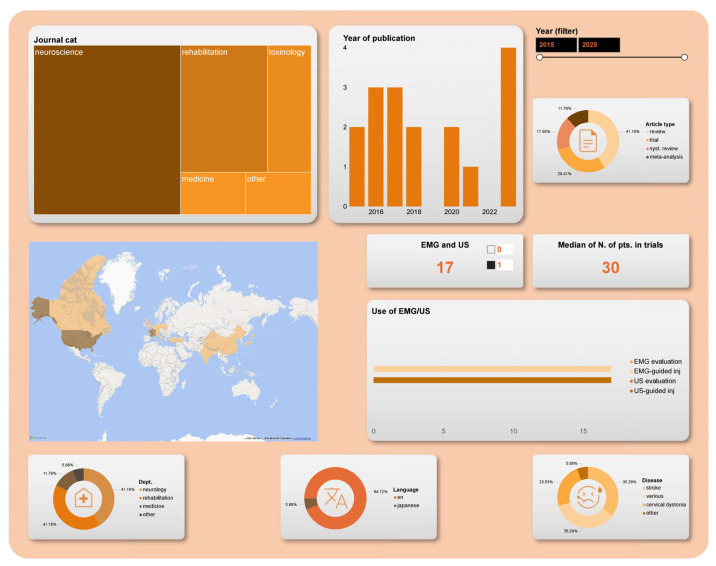
Results of literature reviews considering papers where both EMG and US were used. The variables considered are the same as in Figure 1.

The general characteristics of the selected studies are summarized in Figure 1. For a specific focus on the subset of the literature where both EMG and US were employed, Figure 2 provides a comparative breakdown using the same variables.

The departments most involved in the publications were rehabilitation (38.9%) and neurology (30.5%) [26,27,28,30,31,35,38,39,44,47,48,49,50,51,53,54,57,59,60,61,63,65,66,69,70,71,72,73,77,79]. As expected, most works were written in English (93.2%). The pathologies analyzed most frequently were stroke (20.3% of publications) [30,34,38,43,45,54,57,65,72,73,77,82], cervical dystonia (18.6%) [21,24,32,35,37,44,46,49,63,68,80] and cerebral palsy (CP) (15.3%) [23,25,27,47,52,58,64,69,75]. Multiple pathologies were analyzed without a specific focus on a single disease in 35.6% of the papers [2,28,29,31,36,41,42,48,50,51,53,56,59,61,66,70,76,78,79,81].

Of the 59 works considered, only 12 reported a simultaneous application of EMG and US to guide BoNT injections [2,21,24,30,38,39,43,59,63,77,78,79]. Of these, more than half were published in neuroscience journals, and 2023 accounted for the highest number of publications. Reviews were the most common study (41.2%), followed by clinical trials (29.4%). In terms of geographical origin, works integrating EMG and US were present in both Eurasia and North America. Furthermore, the neurological and rehabilitation departments made the greatest contribution, with an almost exclusive use of the English language. Finally, the pathologies analyzed using this combination were primarily stroke and cervical dystonia. Then, considering exclusively the reviews that included EMG-US interaction, we found a predominance of cervical dystonia, with no reviews related to stroke [21,32,63]. In contrast, stroke was the only disease addressed in clinical trials.

Most of these studies compare US guidance with the anatomical landmark approach [13,79,83]. In all but one of these studies, US guidance demonstrated to be more effective than manual guidance in improving the primary outcome measures [79] (Table 1).

In the remaining study, no significant differences between the two techniques were observed. In this study, EMG guidance was also carried out, but significant differences with both the manual and US approaches were found [79]. However, we found no study evaluating the combination of EMG and US guidance in post-stroke spasticity. Regarding cervical dystonia, we found three studies that compared US guidance with the manual approach [3,74,86]. However, in only one of them, US guidance demonstrated to be more effective than the manual approach by improving both the clinical and the quality-of-life measures [74]. Only two studies compared EMG guidance with the manual approach in CD patients [84,85]. Only one of these two studies has demonstrated that EMG guidance was more effective than the manual approach in improving the primary outcome (modified TWSTRS and patient report) in 52 patients [84]. The second study found no difference between the two treatment groups for almost all of the evaluation sessions, except for week 16, when EMG demonstrated a lower incidence of dysphagia when using EMG guidance for cervical muscle targeting compared to manual injection [85]. Only one study evaluated the combination of EMG and US guidance in patients with cervical dystonia. These authors found that the incidence of dysphagia improved from 35% to 0%, when they switched five patients from the EMG- to the US-guided technique [87]. However, cervical dystonia also appeared in systematic reviews, while stroke was represented in a meta-analysis.

Regarding CP, we found only one study that compared a manual approach with US guidance. In a prospective non-randomized study of 54 patients (30 of whom were injected using US guidance), Py et al. found that the treatment was effective in 51% of children. However, the effectiveness was significantly higher when BoNT/A injections were carried out using US guidance than when the anatomical landmark technique was used. This effect was more evident in younger patients (under 6 years old) [89]. No studies comparing US with EMG guidance, or combining these two approaches, were found. Further, we found no papers on spasmodic dysphonia, dysphagia or cerebral palsy using simultaneous US and EMG guidance for BoNT injections.

## 3. Discussion

This review highlights that the combined use of US and EMG to target BoNT injections remains underrepresented. The profile of the journals, which predominantly cover neuroscience and rehabilitation, reflects the multidisciplinary nature of this approach. It requires expertise in physical medicine and rehabilitation, neurology, and, in some cases, interventional radiology. The geographic distribution, with a greater contribution from Europe and North America, followed by Asia, highlights global interest, as well as a concentration of expertise in reference centers equipped with advanced technological resources. From a clinical perspective, the focus on stroke and cervical dystonia suggests that the technique is perceived as being particularly useful in conditions where the selectivity and precision of injection significantly impact functional outcomes [21,77].

In more detail, both EMG- and US-guided therapy have been shown to be effective in alleviating symptoms in patients with cervical dystonia (CD). In a randomized, double-blind study of 52 patients with CD, Comella et al. demonstrated that EMG guidance was more effective than manual localization in improving the Toronto Western Spasmodic Torticollis Rating Scale (TWSTRS) score, which represented the primary outcome (71% overall improvement) [84].

Conversely, Wu et al. found no superiority of EMG guidance on Tsui scale changes at 4, 8 and 12 weeks after BoNT/A injection compared to the manual approach in 65 patients [85]. However, they found that, compared to the manual approach, EMG guidance showed significant differences in 16 weeks. Furthermore, patients treated with EMG guidance had less dysphagia than those treated manually [85]. In a retrospective study of 75 CD patients analyzed over 1 year, which was primarily aimed at exploring the correlation between BoNT/A injections into the cervical muscles (sternocleidomastoid and scalenes) and dysphagia, US guidance did not reduce the incidence of dysphagia [3].

In a study of 35 patients with CD, Tyślerowicz et al. compared the efficacy of BoNT-A administration using a manual approach with US guidance. They assessed this efficacy using the TWSTRS, the Tsui modified scale, the Craniocervical Dystonia Questionnaire (CDQ-24) and the Clinical Global Impression Improvement scale (CGI-I). Significant decreases in the TWSTRS total score, TWSTRS severity subscale score, Tsui score and CDQ-24 score were observed in both groups. However, a significant decrease in the TWSTRS disability and pain subscales was found only in the US-guided group [74]. Furthermore, US-guided treatment resulted in greater reductions in TWSTRS, Tsui score, and CDQ-24 compared to the manual approach alone. The authors concluded that US guidance might improve the effects of BoNT-A injections in CD by reducing associated pain and disability. However, more studies are needed to evaluate its clinical efficacy [74].

Only one study has assessed the effect of a combined US and EMG approach on dysphagia associated with cervical muscle injections in five CD patients [87]. Using US plus EMG guidance resulted in a complete disappearance of dysphagia, as opposed to EMG guidance alone, which had a cumulative dysphagia rate of 34%. The authors concluded that the combination of US and EMG guidance eliminated dysphagia recurrence following BoNT/A treatment, possibly by preventing the spread of BTX outside the sternocleidomastoid muscle. We did not find any further publications on the use of the US plus EMG combination in CD [87].

Similar to cervical dystonia, several studies on BTX treatment using both US and EMG guidance have demonstrated efficacy in patients with post-stroke spasticity. Picelli et al. compared manual guidance with US guidance and electrical stimulation for BoNT/A injection into the gastrocnemius muscle of 81 adults with spastic post-stroke equinus [83]. The modified Ashworth scale, the Tardieu scale and ankle passive range of motion were measured at baseline and one month after BoNT/A injections. Patients treated with US guidance improved more than those treated with other techniques in terms of the modified Ashworth scale and ankle passive range of motion [83].

Santamato et al. evaluated 30 patients with post-stroke spasticity of the upper limb and compared the efficacy of BoNT/A injection using US guidance or manual needle placement. After one month of follow-up from BoNT/A treatment, spasticity, as measured by the modified Ashworth scale, had significantly improved in both groups. However, the improvement was more significant in patients treated with US guidance than in those injected using the manual approach [13].

In a randomized controlled study of 27 adult patients with hemiplegia and post-stroke spasticity in the upper and lower limbs, the effects of BoNT/A injections using EMG guidance were compared with those using manual approaches. The primary outcome measures, the modified Ashworth Scale (MAS) and the modified Barthel Index were evaluated pre- and post-botulinum toxin injection, with a follow-up period of three months [88]. Three weeks after BoNT/A injection, both the MAS and the modified Barthel Index showed significant improvement in all patients; however, the improvement was greater in patients treated using EMG guidance [88]. Similar results were observed by Py et al. in a study of lower-limb spasticity associated with cerebral palsy. They evaluated 51 children, 30 of whom were injected using US guidance [89]. Overall, BTX was effective in 51% of patients. The efficacy was significantly greater with higher doses, when the injected muscles were gastrocnemius or hamstrings, and when the injections were US-guided [89]. However, we did not find a study assessing the efficacy of BTX injections using a combination of US and EMG in spasticity.

The complete absence of studies on the combined use of US and EMG in other conditions, including cerebral palsy, spasmodic dysphonia, painful conditions associated with abnormal muscular hyperactivity, and cricopharyngeal muscle dysfunction associated with neurogenic dysphagia, indicates significant areas of unexplored research.

The significant proportion of systematic reviews in cervical dystonia and the presence of one meta-analysis in stroke indicates a growing interest in evidence synthesis. However, the limited number of controlled multicenter clinical trials restricts the ability to make robust, generalizable clinical recommendations. Furthermore, this relative scarcity contrasts with the clear advantages of this method, which combines the high-resolution anatomical visualization of US with the functional and neurophysiological confirmation provided by EMG. This synergy undoubtedly improves injection accuracy, minimizes the risk of errors in needle positioning, and optimizes the administered dose—a particularly relevant factor in disorders characterized by anatomical complexity or individual variability [39].

However, even though there are only a few published studies, both US and EMG may be effective in improving the efficacy of BTX injections compared with the manual approach. There is insufficient data definitively demonstrating the superiority of one technique over the other, even though US guidance is more widely used. For this reason, preference for one technique over the other depends largely on experience, familiarity with the method, and personal clinical background. Furthermore, it has not yet been proven that administering BoNT-A guided by EMG and US is more effective than using US alone. Further research in this area is necessary. Intuitively, however, the combination of these two useful techniques can only improve the effectiveness of each technique individually, thereby improving safety. However, due to the absence of comparative studies between the combined approaches of each individual technique, it is not possible to draw definitive conclusions.

Nevertheless, based on our mainly clinical neurophysiological background, we emphasize the added value of EMG, which, due to its peculiarities, goes beyond the effectiveness of US guidance and gives the latter greater precision.

In particular, the added value of EMG guidance to US approach assumes strategic importance when treating conditions characterized by complex, overlapping muscle activation patterns, or when targeting structures that are inaccessible to surface anatomical landmarks and conventional imaging techniques. The rationale behind combining EMG recordings with US guidance goes beyond simple anatomical localization and surpasses the effectiveness of either technique used alone. This is particularly evident in pathological conditions characterized by muscular hyperactivity, especially spasticity and dystonia, where EMG recordings allow identification of the most affected muscle groups and their specific activation behaviors. This is particularly relevant in conditions where it is fundamental to evaluate specific muscle patterns, as for example when injecting the cricopharyngeal (CP) muscle in patients with dysphagia associated with upper esophageal sphincter (UES) dysfunction, where it is crucial to identify the presence and extent of reduced or absent muscular relaxation, or laryngeal dystonia, where EMG can reveal specific dystonic bursting patterns [82,90,91].

Moreover, the combined US/EMG approach may be very useful for targeting deep muscles, including laryngeal, pharyngeal and deep cervical muscles, which are difficult to reach by US guidance alone due to their anatomical location [82,90,91]. Furthermore, in patients with dystonia, EMG enables the differentiation of primary pathological muscle activity from secondary compensatory responses that may not require treatment. Accurate differentiation between these two patterns is essential for targeting optimal treatment, as injecting compensatory muscles instead of primary dystonic muscles can paradoxically worsen functional outcomes while failing to address the primary pathology [8]. For cervical dystonia, this typically includes assessment in a neutral position, during active head turning and during passive positioning whilst requesting muscle relaxation. Primary dystonic muscles demonstrate persistent activity during passive positioning despite relaxation attempts, while compensatory muscles show activation only during active movement attempts and remain silent during passive positioning [8]. Additional evidence supporting the discriminatory capacity of EMG comes from laryngeal dystonia research. Blitzer et al. used laryngeal EMG during phonation in 127 patients with spasmodic dysphonia and identified the primary involvement of the thyroarytenoid muscles in adductor spasmodic dysphonia and the posterior cricoarytenoid muscles in the abductor variant. Crucially, EMG revealed frequent compensatory activation of extrinsic laryngeal muscles (sternothyroid and thyrohyoid) that clinical assessment had incorrectly identified as primary dystonic targets [82].

Another point in favor of the adjunctive value of EMG integration is its ability to facilitate precise needle positioning within the motor endplate region, where acetylcholine receptors are concentrated. EMG-guided injection directly into the endplate zone has already been demonstrated to significantly improve clinical outcomes in spasticity management compared with anatomical landmark techniques, whilst permitting a 30–40% reduction in BoNT dose [92]. The endplate zone can be identified through characteristic EMG signals. As the needle electrode penetrates muscle tissue, there is a marked increase in spontaneous electrical activity upon endplate contact. This produces a distinctive acoustic pattern, described as “endplate noise” or “endplate spikes”, which, when amplified, results in characteristic crackling sounds. These sounds are generated by miniature endplate potentials, which are caused by spontaneous acetylcholine release from motor nerve terminals. This provides real-time confirmation of optimal needle positioning [93]. The dose-sparing effect of endplate targeting has significant clinical and economic implications. In fact, reduced toxin requirements diminish treatment costs and potentially decrease the immunogenicity risks associated with cumulative toxin exposure. They also permit the treatment of additional muscle groups within safe dosing parameters [15,92,93]. For these reasons, even if based on our opinion which of course is dictated by our clinical background, we consider the EMG guidance as essential for botulinum toxin injections.

## 4. Conclusions

Our findings highlight the scarcity of controlled, multicenter clinical trials investigating the combined use of US and EMG for cervical dystonia and stroke, as well as the lack of controlled studies for many of the conditions associated with abnormal muscle hyperactivity in which BoNT has been shown to be effective. In addition, only a few studies have compared the EMG and US approaches, and it is not possible to definitively conclude that one of these techniques is superior to the other. Further studies are needed to clarify this topic. Furthermore, although it has not yet been proven that the combination of BoNT/A administration guided by US and EMG is more effective than using US or EMG alone, further research in this area is necessary. However, the potential development of the US/EMG combination should be considered.

Firstly, expanding the range of conditions for which it is used to include other neurological and muscular disorders, such as cerebral palsy and laryngeal dystonia, or other conditions associated with muscular hyperactivity, could increase the number of patients who are eligible for treatment, offering advantages in terms of procedural accuracy [23]. Secondly, integration with emerging technologies, such as artificial intelligence and the automatic processing of EMG signals and US images, could reduce operator-dependent variability, making the procedure faster and more reproducible [94]. Thirdly, creating multidisciplinary training programs based on simulation, augmented reality and e-learning modules would promote the uniform diffusion of the technique, reduce the learning curve and promote shared quality standards [95]. Additionally, developing international protocols that define the context in which the combined use of US and EMG is preferable to using either method alone would be an essential step towards widespread adoption. Finally, establishing international multicenter registries and conducting cost-effective studies would allow a more precise definition of the clinical and economic impact of combining US and EMG to be defined more precisely, supporting evidence-based decisions and facilitating the inclusion of the method in official guidelines [96].

## 5. Materials and Methods

To perform this literature analysis, a PubMed search was conducted using the following string: “Botulinum toxin AND (spasticity or dystonia) AND (electromyography or diagnostic ultrasound or sonography)”. To identify works of greater scientific relevance, the following filters were used: clinical trials, randomized controlled trials, meta-analyses, guidelines, reviews and systematic reviews. Finally, we considered publications from the last 10 years only. The results were then exported in text format via the Save function on the PubMed website, including titles, abstracts, and editorial information. The abstracts were thus screened to exclude papers that were not relevant to the research topic. For each selected abstract, the following variables were collected: the category of the publication journal (when more than one category was present, the category of greatest significance was considered, based on the quartile ranking to which the journal belonged); the year of publication; the department and country of origin of the first author; the type of work (review, systematic review, meta-analysis, expert opinion or trial); and the pathologies of interest in the work. Additionally, we analyzed the use of US and EMG, whether used separately or simultaneously, and whether for evaluation purposes or to guide treatment with botulinum toxin. Finally, the number of patients in each clinical trial was considered and expressed as a median value. All collected data were then entered into a database and imported into Power BI (version 2.153.910.0), a data analysis software able to summarize the results using dynamic graphs. This method can show data filtered by specific variables. Depending on the selection made by the data analyst, subpopulations of results can therefore be displayed.

The following graphs were used: a tree map for the journal categories, bar graphs for the year of publication, grouped bar graphs to count the uses of EMG and US, a geographical map to show the distribution of works across various territories, and doughnut graphs to show the percentages of article types, production departments, languages used, and considered pathologies. Then, works using US and EMG simultaneously to guide BoNT injections were selected using a custom filter. Finally, the median number of patients for clinical studies was shown using a simple data card. Thus, the system can provide information by selecting specific variables. For example, it is possible to select the review variable for the type of articles and display all the other collected characteristics related to that type of article. This allows for precise displays based on the questions asked. This approach allows precise data explorations based on specific research questions. We focused on presenting the simultaneous use of EMG and US methods for BoNT injection guidance. We then made a specific comparison between the general results and the results related to works involving the simultaneous application of these two techniques. Furthermore, these works were filtered by article type, with a comparison between primary and secondary articles.

## Figures and Tables

**Table 1:** Comparison among different botulinum toxin injection methods in different neurological diseases.

	Disease	Patient Sample	EMG	US	Manual	Results
Comella et al. [84]	CD	52	Y	N	Y	EMG better than manual
Wu et al. [85]	CD	65	Y	N	Y	EMG better than manual at 16 weeks
Kutshenko et al. [3]	CD	75	N	Y	Y	No differences in dysphagia reduction
Tyślerowicz et al. [74]	CD	35	N	Y	Y	More improvement using US in TWSTRS
Kreisler et al. [86]	CD	123	N	Y	Y	US better than manual
Hong et al. [87]	CD	5	Y	Y	N	EMG + US better dysphagia reduction
Picelli et al. [83]	Spasticity	81	N	Y	Y	US better than manual
Santamato et al. [13]	Spasticity	30	N	Y	Y	US better than manual
Ploumis et al. [88]	Spasticity	27	Y	N	Y	EMG better than manual
Py et al. [89]	CP	51	N	Y	Y	US better than manual

CD: cervical dystonia, CP: cerebral palsy, EMG: electromyography, N: no, TWSTRS: Toronto Western Spasmodic Torticollis Rating Scale, US: ultrasound, Y: yes.

## Data Availability

No new data were analyzed on this study.

## Abbreviations

The following abbreviations are used in this manuscript:

BoNT	Botulinum neurotoxin
CD	Cervical dystonia
CP	Cerebral palsy
EMG	Electromyography
MAS	Modified Ashworth Scale
TWSTRS	Toronto Western Spasmodic Torticollis Rating Scale
US	Ultrasound
